# Untargeted lipidomics reveal association of elevated plasma C18 ceramide levels with reduced survival in metastatic castration-resistant prostate cancer patients

**DOI:** 10.1038/s41598-023-44157-9

**Published:** 2023-10-18

**Authors:** Carlo Cattrini, Marcello Manfredi, Paola Barboro, Marco Ghirimoldi, Alessia Mennitto, Veronica Martini, Alessio Battioni, Marco Le Van, Simone Gobbato, Carmen Branni, Rahma Ben Ayed, David James Pinato, Fabio Catalano, Elisa Zanardi, Francesco Boccardo, Alessandra Gennari

**Affiliations:** 1 Department of Internal Medicine and Medical Specialties (DIMI), School of Medicine, University of Genoa, 16132 Genoa, Italy; 2 Medical Oncology, “Maggiore Della Carità” University Hospital, 28100 Novara, Italy; 3 Department of Translational Medicine, University of Piemonte Orientale (UPO), 28100 Novara, Italy; 4 Center for Translational Research on Autoimmune and Allergic Diseases (CAAD), University of Piemonte Orientale, 28100 Novara, Italy; 5 UO Clinica Di Oncologia Medica, IRCCS Ospedale Policlinico S. Martino, 16132 Genova, Italy; 6 Department of Surgery and Cancer, Imperial College London, Hammersmith Hospital Campus, London, UK

**Keywords:** Cancer, Prostate cancer, Lipidomics

## Abstract

Emerging evidence highlights the potential prognostic relevance of circulating lipids in metastatic castration-resistant prostate cancer (mCRPC), with a proposed 3-lipid signature. This study aims to analyze the lipidomic profiles of individuals with mCRPC to identify lipid species that could serve as predictive indicators of prognosis and therapeutic response. Plasma samples were collected from mCRPC patients initiating first-line treatment (1 L) (n = 29) and those previously treated with at least two lines of therapy (> 2 L) (n = 19), including an androgen-receptor signaling inhibitor and a taxane. Employing an untargeted lipidomic approach, lipids were extracted from the plasma samples and subjected to analysis. A comprehensive identification and quantification of 789 plasma lipids was achieved. Notably, 75 species displayed significant dysregulation in > 2 L patients in comparison to the 1 L group. Among these, 63 species exhibited elevated levels, while 12 were reduced. Patients included in > 2 L cohort showed elevated levels of acylcarnitines (CAR), diacylglycerols (DG), phosphatidylethanolamines (PE), triacylglycerols (TG), and ceramides (Cer). Notably, some upregulated lipids, including CAR 14:0, CAR 24:1, Cer d18:1/16:0, Cer d18:1/18:0 (C18 Cer), Cer d18:2/18:0, Cer d18:1/24:1, and Cer d20:1/24:1, showed significant associations with overall survival (OS) in univariate models. Specifically, increased levels of C18 Cer remained significantly associated with poorer OS in the multivariate model, even after adjusting for treatment line and PSA levels (Hazard Ratio: 3.59 [95% Confidence Interval 1.51–8.52], p = 0.004). Employing quantitative mass spectrometry, our findings underscore the independent prognostic significance of C18 Cer in individuals with mCRPC. This discovery opens avenues for further studies within this field.

## Introduction

Metastatic castration-resistant prostate cancer (mCRPC) represents a clinical challenge due to its lethality, primarily attributed to a combination of factors, including resistance to androgen-deprivation therapy, widespread metastatic dissemination, aggressive tumor behavior, and a paucity of effective treatment modalities. Advances in research and treatment have led to a growing array of therapeutic options, including chemotherapy, new hormonal agents and radiopharmaceuticals^1^. The primary limitation of these treatments is the early development of resistance that occurs through several mechanisms^2^. It is therefore an unmet clinical need to identify prognostic and predictive biomarkers of response to therapies.

Current data support the notion that advanced prostate cancer (PCa) is characterized by metabolic reprogramming and dysregulated lipid metabolism^3–7^. Metabolic reprogramming can occur as a consequence of overexpression of oncogenes or deficiency of tumor suppressor genes. However, phenotypic plasticity may intervene through mechanisms of epigenetic dysregulation and/or signals from the tumor microenvironment, even in the absence of specific gene alterations^8^. It has been shown that PCa cells show increased lipid lipogenesis and lipolysis, as well as altered metabolism of cholesterol and phospholipids^9,10^. A strict relationship between androgen-receptor variant 7 (AR-V7) and reactivation of lipid synthesis in CRPC was identified, suggesting the existence of an association between lipid metabolism and the development of resistance to androgen-receptor signaling inhibitors (ARSi)^4,11,12^.

In-depth delineation of lipid metabolism in PCa is therefore significant to open new insights into PCa natural history and resistance to therapies and provide new predictive biomarkers. Many researchers have explored the ability of specific lipid species to serve as biomarkers for the diagnosis of PCa, and some data regarding the potential prognostic and predictive role of specific lipid species during treatment with ARSi or chemotherapy are also available^13–16^.

An Australian group undertook comprehensive plasma lipidomic profiling in patients with metastatic castration-resistant prostate cancer (mCRPC), demonstrating that higher levels of ceramide, acylcarnitine and sphingomyelin species are associated with shorter overall survival (OS)^17,18^. These authors also identified a prognostic 3-lipid signature, including ceramide d18:1/24:1, sphingomyelin d18:2/16:0 and phosphatidylcholine 16:0/16:0. This signature was associated with poor outcome at different disease stages, and elevated circulating sphingolipids, especially ceramides (Cer), were associated with specific genomic alterations in mCRPC^18,19^. A recent multi-omic analysis has also confirmed the relevance of Cer in predicting prognosis in patients with PCa^20^.

In this study, we explored the prognostic significance of lipid species in patients with mCRPC using an untargeted lipidomic approach. In contrast to previous studies, we initially compared two mCRPC patient populations to identify the most highly expressed lipids in multi-drug resistant patients compared to those in a more favorable clinical stage. We then assessed the prognostic value of the lipids identified through this comparison. We also tested the prognostic value of lipid species included in the previously reported 3-lipid signature in our patient cohort.

## Materials and methods

### Sample collection and patient population

Patients were eligible for inclusion in this study if they showed mCRPC disease, according to the Prostate Cancer Clinical Trials Working Group 3 (PCWG3) and RECIST 1.1 criteria^21^. Cohort 1L included patients who were starting first-line treatment for mCRPC, including abiraterone acetate, enzalutamide, or docetaxel. Cohort > 2L included patients who had already received at least two lines for mCRPC (including abiraterone and/or enzalutamide and one taxane regimen); these patients could have been candidates for active or palliative treatment with abiraterone acetate, cabazitaxel, docetaxel, cyclophosphamide, enzalutamide, mitoxantrone, or vinorelbine.

Infored consnt was obtained from all patients and/or their legal guardians after approval of the study protocol by the “Ospedale Policlinico S. Martino” Ethics Committee (P.R. 505REG2015—comitato.etico@hsanmartino.it). All research was performed in accordance with relevant guidelines/regulations and in accordance with the Declaration of Helsinki.

Patients with mCRPC managed at the IRCCS Policlinico San Martino Hospital in Genoa were invited to participate in this study. Patients had a blood draw and were prospectively followed up with PSA assessments every 4–6 weeks until death or a cutoff date of December 31, 2018. The first patient was enrolled in October 2016 and a survival update was performed in June 2022. For each patient, 2 mL of blood were collected in vacuum tubes containing EDTA and were centrifuged at 3000×*g* for 10 min. Plasma was obtained by supernatant centrifugation at 15,000×*g* for 10 min and aliquots were stored at − 80 °C.

### Sample preparation

Unique anonymized codes have been assigned to the samples for processing and subsequent analysis, maintaining the confidentiality of personal data. The extraction of plasma lipids was carried out with a biphasic method: 30 µL of plasma was introduced into a tube and extracted with 225 µL of cold MeOH containing a combination of deuterated standards (Splash Lipidomix®). The solution was then vortexed for 10 s, and 750 µL of cold MTBE was added and vortexed for 10 s. The tube was then placed in a thermomixer at 4 °C and vortexed for 6 min at 2000 rpm. After that, 188 µL of water was added, and the tube was vortexed for 10 s and then centrifuged for 2 min at 14,000 rpm at 4 °C. Finally, 300 µL of supernatant was collected and evaporated with a SpeedVac. The dried sample was replenished with 50 µL of a 9:1 MeOH/toluene solution containing the internal standard CUDA (12.5 ng/mL).

### Liquid chromatography–mass spectrometry analysis

Reconstituted samples were analyzed with a Vanquish UHPLC system (Thermo Scientific, Rodano, Italy) coupled with an Orbitrap Q-Exactive Plus (Thermo Scientific, Rodano, Italy). Lipid separation was performed using a reversed-phase column (Hypersil Gold™ 150 × 2.1 mm, particle size 1.9 µm) maintained at 45 °C with a flow rate of 0.260 mL/min. Mobile phase A for ESI mode positive consisted of 60:40 (v/v) acetonitrile/water with ammonium formate (10 mmol) and 0.1% formic acid, while mobile phase B was 90:10 isopropanol/acetonitrile (v/v) with ammonium formate (10 mmol) and 0.1% formic acid, while in the negative ESI mode, the organic solvents for both mobile phases were the same as in the positive with the exception of using ammonium acetate (10 mmol) as a mobile phase modifier. The gradient used was as follows: 0–2 min from 30 to 43% B, 2–2.1 min from 43 to 55% B, 2.1–12 min from 55 to 65% B, 12–18 min at 65% to 85% B, 18–20 min at 85% to 100% B; 100% B was held for 5 min, and then the column was allowed to equilibrate to 30% B for another 5 min. The total running time was 30 min.

Mass spectrometry analysis was performed in both positive ion (at 3.5 kV) and negative ion (2.8 kV) modes. Data were collected in a data-dependent top 10 scan mode (ddMS2). MS full-scan survey spectra (mass range m/z 80–1200) were acquired with a resolution of R = 70,000 and target AGC of 1 × 10^6^. MS/MS fragmentation was performed using high energy c-trap dissociation (HCD) with R = 17,500 resolution and 1 × 105 AGC target. The step normalized collision energy (NCE) was set to 15, 30 and 45. The injection volume was 3 µL. For accurate mass-based analysis, regular Lockmass and interrun calibrations were used. An exclusion list for background ions was generated by testing the same procedural sample for both positive and negative ESI modes^22^.

Quality control was ensured by analyzing pooled samples before, at the beginning and at the end of the batches; using blanks to check for residual interference; and using internal standards, directly in plasma samples, which include a series of analyte classes at levels appropriate for the plasma (Avanti SPLASH Lipidomix) and an internal standard (CUDA) prior to liquid chromatography-mass spectrometry (LC–MS) analysis.

### Data processing

Raw data acquired from untargeted analysis were processed with MSDIAL software (Yokohama City, Kanagawa, Japan), version 4.24. Peaks were detected, MS2 data were deconvoluted, compounds were identified, and peaks were aligned across all samples. For quantification, the peak areas for the different molecular species detected were normalized using the deuterated internal standard for each lipid class. To obtain an estimated concentration expressed in nmol/mL (plasma), the normalized areas were multiplied by the concentration of the internal standard. An in-house library of standards was also used for lipid identification. MetaboAnalyst 4.0 software (www.metaboanalyst.org) was used for the statistical analysis.

### Statistical analysis and biomarker cut-points

X-Tile was used to optimize the outcome-based cut-point and to identify lipid species whose plasma values increased or decreased proportionally with the hazard risk of OS^23^. Survival curves were constructed with the Kaplan‒Meier method and then compared with the log-rank test. Variables with significant prognostic effects were entered into multivariate Cox models to explore the independent prognostic effect of specific lipid species. Biochemical response was defined as a 50% or greater decrease from baseline PSA values^21^. Descriptive statistics were employed to evaluate the response to treatments based on circulating levels of specific lipid species.

## Results

### Patient characteristics

Overall, a total of 48 mCRPC patients had available plasma samples for this analysis, including 29 patients in the 1 L cohort and 19 patients in the > 2 L cohort. Baseline patient characteristics are described in Table 1.

**Table 1:** Baseline patient characteristics.

Variables	1 L N = 29	> 2 L N = 19	Total N = 48
Median age, years (range)	76 (56–84)	70 (58–84)	74 (56–84)
PSA, ng/mL			
< 100	26 (90%)	7 (37%)	33 (69%)
≥ 100	3 (10%)	12 (63%)	15 (31%)
Bone metastases			
Absent	7 (24%)	3 (16%)	10 (21%)
Present	22 (76%)	16 (84%)	38 (79%)
Visceral metastases			
Absent	26 (90%)	13 (68%)	39 (81%)
Present	3 (10%)	6 (32%)	9 (19%)
Number of metastatic sites			
= 1 site	11 (38%)	6 (32%)	17 (35%)
> 1 site	18 (62%)	13 (68%)	31 (65%)

1 L = patients starting first-line treatment for mCRPC; > 2 L = pretreated patient cohort.

### Discovery lipidomic analysis (> 2 L vs. 1 L patients)

**Figure 1:**
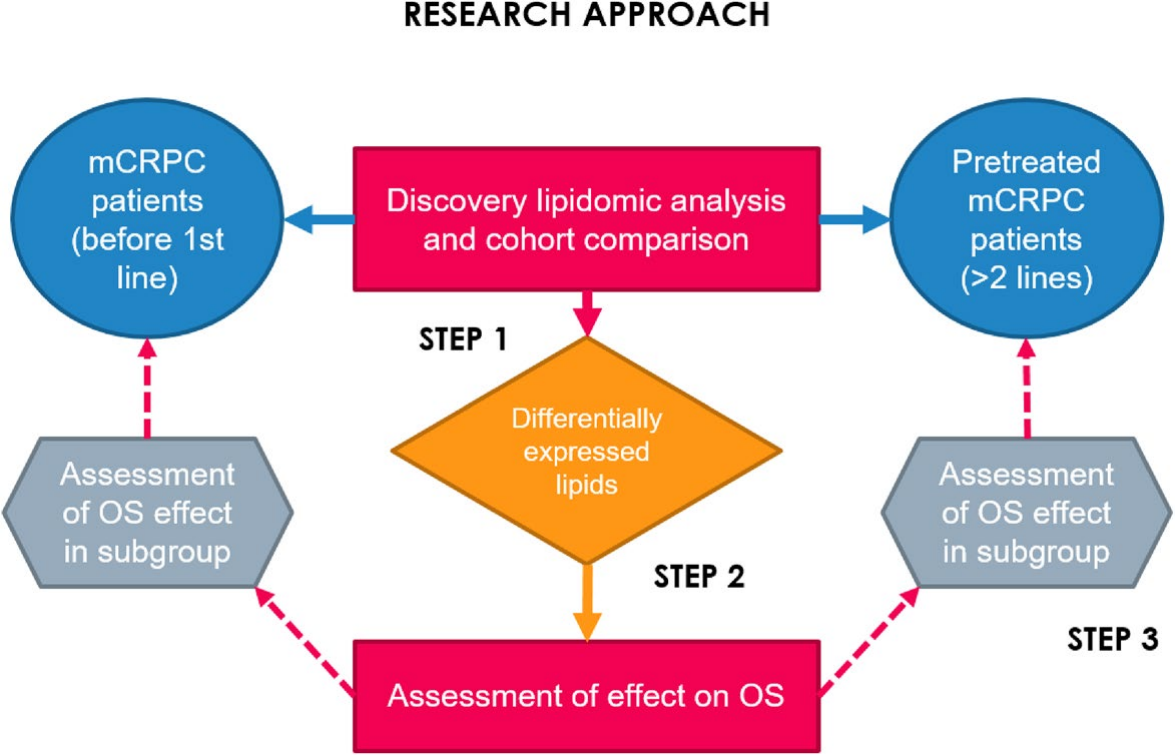
Research approach of this lipidomic analysis in patients with metastatic castration-resistant prostate cancer (mCRPC). An untargeted discovery lipidomic analysis was conducted in two cohorts of patients, including men who were going to start a first-line treatment for mCRPC (1 L cohort) and those pretreated with at least two lines of treatment (> 2 L). Lipids differentially expressed between these cohorts were then tested for their effect on overall survival (OS) using X-Tile to identify appropriate cutoff points. Lipids with a proportional increased risk of death were then tested in multivariate analysis and in both cohorts separately (subgroup analysis).

Using LC‒MS/MS, a total of 789 circulating lipids were quantified in the plasma of the 48 patients involved in this analysis. As explained in Fig. 1, we initially compared the lipidomic profile of pretreated (> 2 L) mCRPC patients with those initiating first-line treatment (1 L).

**Figure 2:**
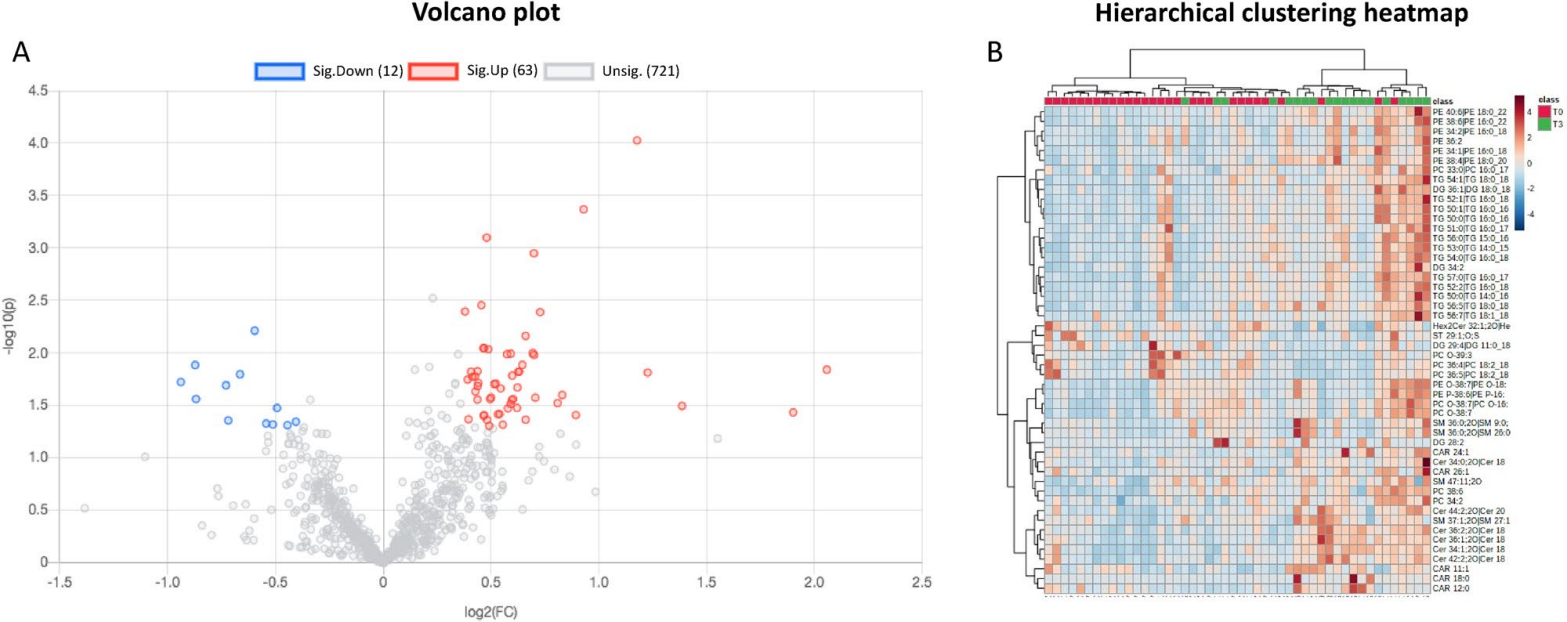
Lipid species differentially expressed in > 2 L compared to 1 L patients. (**A**) Volcano plot reporting overexpressed lipids (in red), downregulated lipids (12, in blue), and nonsignificant lipids (in gray) in > 2 L vs. 1 L. Lipids overexpressed had a fold change > 1.2 and p value < 0.05, whereas dowregulated lipids had a fold change < 0.8 and p value < 0.05. (**B**) Heatmap of the 68 differentially expressed lipids. In the first row, red squares indicate patients belonging to the 1 L cohort, whereas green squares indicate patients belonging to the > 2 L cohort.

The volcano plot in Fig. 2A shows the differential levels between the > 2 L and 1 L cohorts. The red dots identify overexpressed lipids in the > 2 L cohort, whereas blue dots represent the underexpressed lipids in the same cohort. Sixty-three lipids were overexpressed with a fold change > 1.2 and p value < 0.05 (Table 1 Supp), whereas 12 were downregulated with a fold change < 0.8 and p value < 0.05 (Table 2 Supp).

The heatmap in Fig. 2B shows that lipid species overexpressed in the > 2 L group were acylcarnitines (CAR) triacylglycerols (TG), sphingomyelins (SM), diacylglycerols (DG), phosphatidylethanolamines (PE), phosphatidylcholines (PC) and Cer.

### Explorative analysis to assess the association of lipid species with prognosis

After identification of deregulated lipids in the 2 L vs. 1 L cohort, we used X-Tile to optimize the outcome-based cut-point and to identify lipid species whose plasma values increased or decreased proportionally with the hazard risk of OS^23^. Of 49 patients enrolled, 47 had adequate clinical and follow-up data available (1 L n = 29, > 2 L n = 17). Among baseline characteristics, PSA levels and line of treatment (1 L vs. > 2 L) were identified to significantly impact patients’ OS in univariate analysis. Median follow-up for OS was 60.6 months.

Among deregulated lipids, we found that plasma values of CAR 14:0, CAR 24:1, Cer d18:1/16:0, Cer d18:1/18:0 (C18 Cer), Cer d18:2/18:0, Cer d18:1/24:1 and Cer d20:1/24:1 increased proportionally with the risk of death and were significantly associated with OS in univariate analysis using an appropriate cut-point (detailed analysis in Supplementary File 1). However, Cer d18:1/16:0, Cer d18:2/18:0, Cer d18:1/24:1 and Cer d20:1/24:1 did not retain a statistically significant association with OS in the multivariate models (including PSA levels and line of treatment) and were not further investigated. Plasma values of underexpressed lipids did not show a proportional association with OS using X-Tile and were not tested in multivariate models.

### Lipids of the 3-lipid signature and association with clinical outcome

We explored the association with clinical outcome of 3 lipids included in the previously validated 3-lipid signature, namely, ceramide d18:1/24:1, sphingomyelin d18:2/16:0 and phosphatidylcholine 16:0/16:0^17,18^.

As described above, using a cutoff of 31 ng/mL, we found that high levels of Cer d18:1/24:1 were associated with poor OS in the univariate model. The median OS was 7.2 months (95% CI 3.3–11.0) in patients with high levels and 24 months (95% CI 2.2–45.8) in those with low levels (p = 0.009). However, no statistically significant association with OS was found after adjustment for treatment line and baseline PSA (HR 1.54, 95% CI 0.74–3.2, p value = 0.25). We did not find any association between plasma values of sphingomyelin d18:2/16:0 or phosphatidylcholine 16:0/16:0 and OS.

### Association of Cer C18 Cer with survival

**Figure 3:**
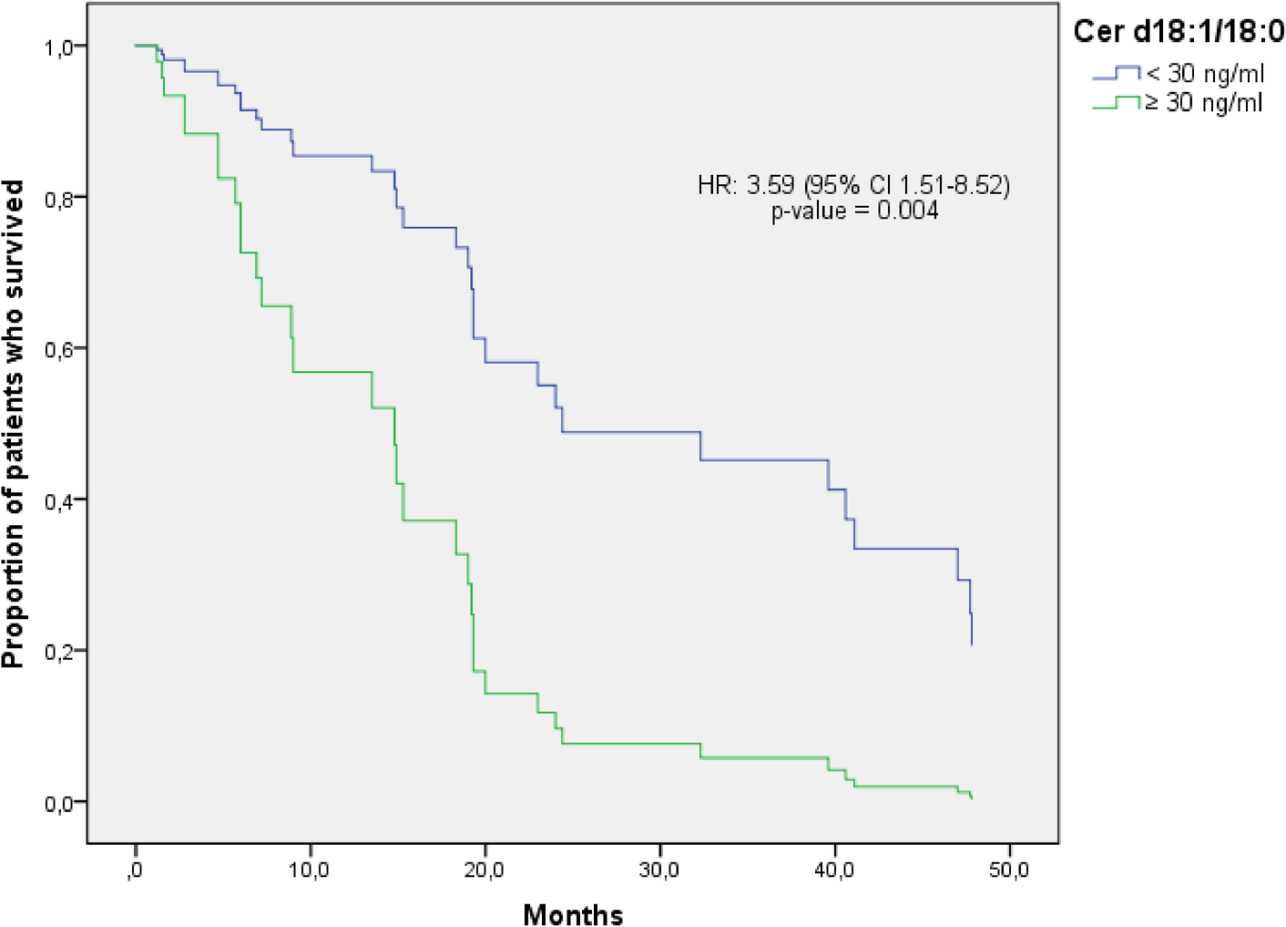
OS multivariate analysis of C18 Cer. This figure shows Kaplan–Meier curves using Cox regression to adjust for basal PSA levels and line of treatment. The green line represents patients with C18 Cer ≥ 30 ng/ml, whereas the blue line represents patients with C18 Cer < 30 ng/ml.

Cer d18:1/18:0 (C18 Cer) was the only lipid species associated with prognosis in univariate analysis that retained statistical significance after adjustment for significant prognostic variables. X-Tile identified an ideal cutoff of 30 ng/mL. Patients with higher C18 Cer plasma values showed a significantly increased risk of death compared to those with lower values (HR: 3.59 [95% CI 1.51–8.52] p = 0.004), irrespective of basal PSA levels and line of treatment (Fig. 3). The median OS was 6.9 months (CI 95%, 4.5–9.2) in patients with high C18 Cer plasma values compared to 40.6 months (CI 95%, 12.8–68.3) in those with low levels.

**Figure 4:**
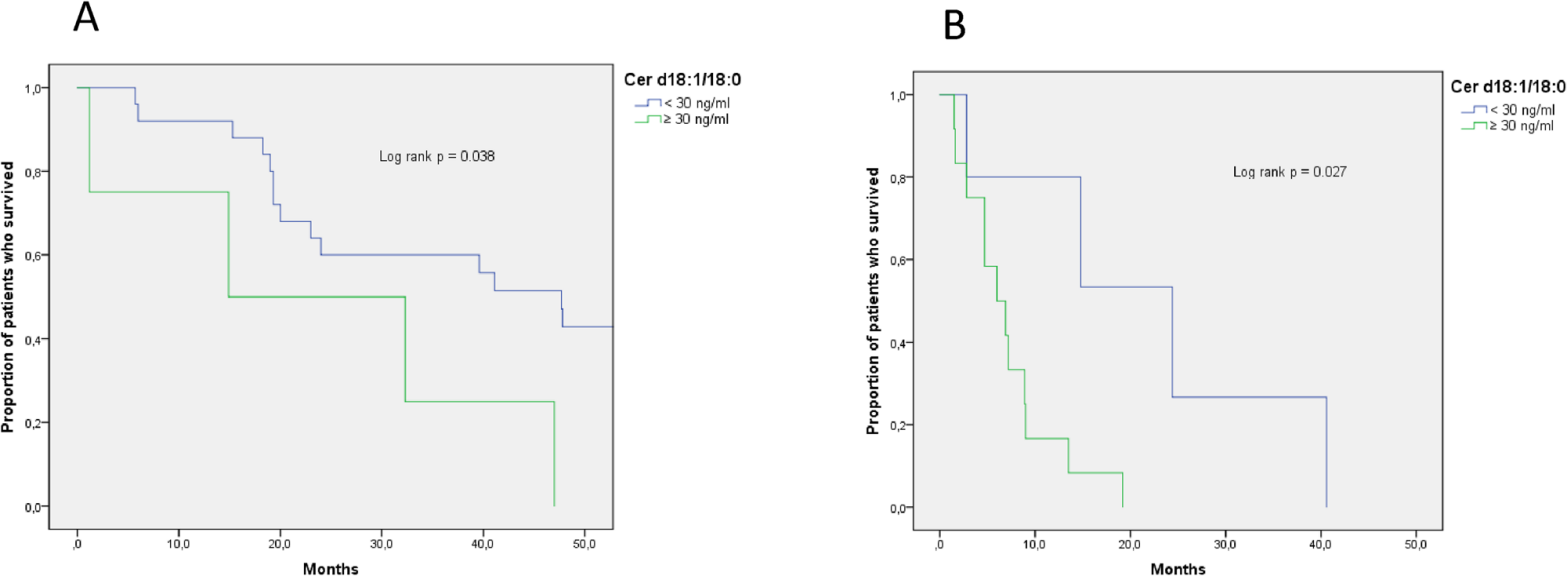
OS subgroup analysis of C18 Cer in the 1 L (**A**) and > 2 L (**B**) cohorts. Univariate unadjusted models were used. The green line represents patients with C18 Cer ≥ 30 ng/ml, whereas the blue line represents patients with C18 Cer < 30 ng/mL.

Of interest, the subgroup analysis confirmed an unfavorable association between C18 Cer levels and patient survival in both the 1 L and > 2 L cohorts separately considered (Fig. 4). In the 1 L cohort, the median survival was 14.9 (CI 95%, 0–45.4) months compared to 47.7 (CI 95%, 35.0–60.3) months for subjects with high (n = 4) vs. low (n = 25) C18 Cer levels (p value = 0.038). In the > 2 L group, patients with high C18 Cer levels (n = 12) showed a median survival of 6 months (CI 95%, 2.3–9.7) compared to 24.4 months (CI 95%, 6.5–42.3) for those with low levels (n = 5) (p value = 0.027).

### Association of C18 Cer with PSA response

An explorative correlation between plasma C18 Cer levels and response to treatment was also evaluated. Response to treatment was assessed through the achievement of PSA50 (50% or greater reduction in PSA values). Overall, 19 of 28 patients (67.9%) who had low C18 Cer levels achieved PSA50; conversely, only 6 out of 14 patients (42.9%) who had high C18 Cer levels achieved PSA50. Of patients who initiated first-line ARSi, 1 of 2 with elevated C18 Cer levels achieved PSA50. Of the two patients who started first-line docetaxel with elevated C18 Cer levels, 2 achieved PSA50.

### Combination of C18 Cer and CTC positivity

**Figure 5:**
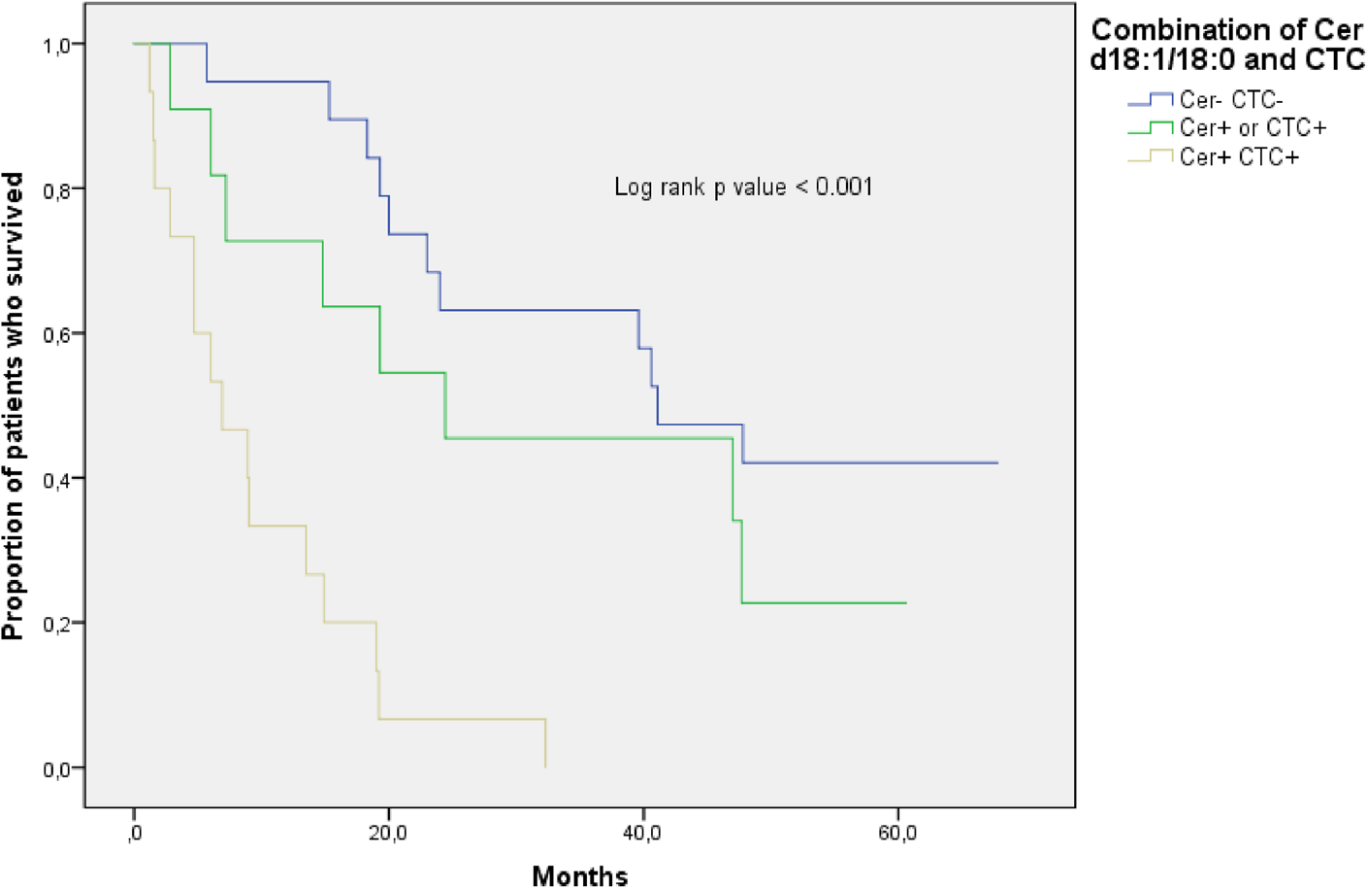
Explorative prognostic model including C18 Cer and CTC positivity. Kaplan Meier curves and log-rank test are shown. The blue line represents patients with C18 Cer < 30 ng/mL and CTC-negative, the green line represents patients with C18 Cer ≥ 30 ng/mL or CTC-positive, the yellow line represents patients with C18 Cer ≥ 30 ng/mL and CTC-positive.

We finally explored the prognostic effect on OS of combining plasma values of C18 Cer levels and circulating tumor cell (CTC) positivity, which was previously assessed in the same cohorts of patients^24^. We investigated a model that included three categories of patients (CTC− Cer−; Cer+ or CTC+; Cer + CTC +). The median OS of the entire population was 41.1 months (CI 95%, 29.4–52.8) for Cer− CTC− (n = 20), 24.4 months (CI 95% 0–56.5) for Cer + or CTC + (n = 11), and 6.9 months (CI 95%, 1.6–12.2) for Cer + and CTC + (n = 15) (Fig. 5).

## Discussion

Despite recent therapeutic advances, the prognosis of patients with metastatic PCa remains poor^25^. Multiple prognostic and predictive factors have been investigated to identify patients with PCa at increased risk of progression or death. In the case of mCRPC, only alterations in DNA damage and response genes, in particular BRCA1 and BRCA2, have been validated to guide therapeutic choices^26,27^. Therefore, there remains a clinical unmet need to identify new prognostic and predictive factors. Some studies have already been conducted to assess the levels of lipid species in patients affected by mCRPC to explore their prognostic and predictive significance. In this regard, relevant studies were published by an Australian research group^17–19,28^. These authors proposed a prognostic three-lipid signature that included ceramide d18:1/24:1, sphingomyelin d18:2/16:0 and phosphatidylcholine 16:0/16:0.

Our study involved 48 patients with mCRPC who were going to start a first-line treatment for mCRPC (1 L cohort – n = 29) or who had already received two or more lines of treatment for mCRPC (> 2 L cohort—n = 19). Unlike earlier research^17–19,28^, we conducted a comparative analysis of two mCRPC patient groups to pinpoint the lipids with the highest expression levels in multi-drug resistant patients compared to those in a more favorable clinical condition. A total of 789 lipids were analyzed and identified, and a comparison between lipid levels in the 1 L and > 2 L cohorts was performed. From this preliminary investigation, we identified 63 overexpressed lipids in the > 2 L cohort and 12 downregulated lipids. At the level of lipid classes, > 2L patients showed higher levels of CAR, DG, PC, TG, SM, PE, Cer and TG. Among underexpressed lipids, we identified specific PC and DG species. These results are mostly consistent with literature data: several findings have confirmed the differential expression of CAR, DG, SM, PC and Cer species among individuals with PCa, as well as and their association with clinical outcome^17–20,28^.

Among all deregulated lipids, we found that high plasma levels of CAR 14:0, CAR 24:1, Cer d18:1/16:0, Cer d18:1/18:0 (C18 Cer), Cer d18:2/18:0, Cer d18:1/24:1 and Cer d20:1/24:1 showed a proportional increased risk of death using X-Tile and a significant association with OS in univariate models. Of note, Cer d18:1/16:0, C18 Cer, and Cer d18:1/24:1 (the latter included in the 3-lipid signature) were previously validated for their prognostic significance in mCRPC patients^17,18^. In addition, C18 Cer and Cer d18:1/24:1 were recently associated with poor survival in patients with mCRPC in another multiomic analysis^20^. Furthermore, CAR species ranked among the top 20 significant lipids associated with shorter OS in the mCRPC cohort reported by Lin and colleagues^18^; CAR are mediator of cancer metabolic plasticity, migration and invasion in PCa cells^29^. Therefore, the results of our discovery analysis are consistent with prior studies. We did not identify any association between OS and the other two lipids individually analyzed, which were included in the 3-lipid signature, namely, sphingomyelin d18:2/16:0 and phosphatidylcholine 16:0/16:0. However, we were not able to test the 3-lipid signature using the formula proposed^17^, given the lack of standardized normalization. Therefore, we cannot conclude whether this signature might be able to stratify our patients.

In our cohort, C18 Cer was the only lipid species that retained a statistically significant association with patient OS after adjustment for basal PSA and line of treatment in the multivariate model. While numerous prognostic indicators exist, the primary prognostic factors of our small population were determined to be the treatment line and initial PSA values. This result is unsurprising and aligns with existing knowledge in the medical literature^1^. The sustained statistical significance of C18, even after adjusting for these potential confounding factors, underscores its relevance as an independent prognostic factor. This ceramide was not included in the 3-lipid signature, but it was reported among the individual prognostic lipids in the study performed by Lin and colleagues^17^. In our study, patients with higher levels of C18 Cer showed a significantly increased risk of death compared to those with lower levels (HR: 3.59 [95% CI 1.51–8.52], p = 0.004), with a median OS of 6.9 months (CI 95% 4.5–9.2) in patients with high plasma values compared to 40.6 months (CI 95% 12.8–68.3) in those with low levels. Of significant interest, the subgroup analysis confirmed an unfavorable association between C18 Cer levels and patient OS in both the 1 L and > 2 L cohorts, separately analyzed. This observation is relevant in that it is inconsistent with a possible selection bias related to initial lipid selection during cohort comparison (Step 1: 1 L vs. > 2 L): C18 Cer was selected in highly pretreated patients with expected reduced survival, but it retained prognostic value in 1 L patients.

With regard to PSA response, we observed that 42.9% of patients with high C18 Cer levels achieved PSA50, compared to 67.9% with low ceramide levels. However, given the heterogeneity of treatments and patient characteristics, as well as the small sample size, further investigations are needed to explore the predictive value of this ceramide.

We also explored the prognostic potential of a nomogram that included C18 Cer and CTC positivity. Although this nomogram should be considered exploratory, it supports the additional value of this ceramide to current known prognostic biomarkers.

Overall, our data are consistent with the observation that elevated circulating Cer levels are associated with poor outcomes in patients with mCRPC^17–19^. Classically, ceramides have anti-tumorigenic functions, inducing senescence and growth inhibition in cancer. However, some studies suggest that ceramide effects are context dependent and rely on downstream effectors, which can both promote or inhibit tumor growth^30^. Depending on the length of their acyl side chain, all ceramides can be grouped as long-chain (C14:0-C20:0), very-long-chain (C22:0-C26:0) and ultra-long-chain (> 26 carbons). Different ceramide length often results in distinct biological activity. In our study, we found that both long- and very-long-chain ceramides might have prognostic significance.

Cer metabolism is quite complex and involves three major pathways^31^. (1) Cer can be generated via a de novo route occurring within the endoplasmic reticulum, which encompasses a series of enzymatic reactions, including the involvement of ceramide synthase (CerS). (2) Cer can be created through the conversion of sphingomyelin (SM) by the action of sphingomyelinase (SMase). 3) The salvage pathway generates Cer from sphingosine, a product of complex sphingolipid metabolism via CerS. Once produced, Cer can undergo conversion into ceramide-1-phosphate (C1P) facilitated by the enzyme ceramide kinase (CERK), or can be transformed back into sphingosine by acid ceramidase (AC). Subsequently, sphingosine is phosphorylated by sphingosine kinases (SPHK) to give rise to sphingosine-1-phosphate (S1P). Several literature data support the involvement of CerS, CERK and SPHK in PCa biology^32,33^, and some studies suggest that enhanced ceramide-S1P signaling may mediate ARSi resistance induced by AR gain^34^.

Of interest, aberrant Cer metabolism in PCa could be modulated by targeting the metabolic environment of the host. Cardiovascular and obesity studies demonstrate that elevated circulating Cer can be decreased using cholesterol-lowering drugs (statins and PCSK9 inhibitors) and exercise^35–37^. The therapeutic effects of statins have been widely investigated in patients with PCa^38^, and a recent study demonstrated that a negative profile of 3-lipid signature (including high levels of ceramides) can be reversed using simvastatin in patients with mCRPC^28^.

In addition, preclinical evidences suggest that pharmacological inhibition of SPHK can effectively reduce CRPC cell proliferation and xenograft tumor growth by targeting AR and the oncogene MYC^39^. In vitro experiments also showed that de novo resistance to enzalutamide in androgen-independent cells can be reversed with SPHK inhibitors in vitro^34^. SPHK inhibitors are being tested in patients with cancer in phase I and phase II trials^40^.

Ceramides are also activators of PLA2, an enzyme that releases arachidonic acid for subsequent conversion to prostaglandins, molecules involved in inflammation, immunity, and tumor growth modulation. Increased levels of prostaglandins, such as PGE2, are associated with enhanced PCa proliferation and invasion, which can be reversed by the use of cyclooxygenase (COX) inhibitors^41,42^.

In light of all these results, further studies are needed to explore the therapeutic targeting of Cer in patients with mPCa. Proper patient selection is probably the key to success, and C18 Cer plasma levels might help identify patients with impaired Cer metabolism.

### Strengths and limitations

This is the first translational study to provide clear evidence that C18 Cer could serve as a useful prognostic biomarker in patients with mCRPC. For the first time, our analysis showed that this ceramide has independent prognostic value after adjustment for relevant confounding factors in patients with mCRPC. In addition, this Cer might help in identifying patients suitable for treatments that target lipid metabolism. Our hierarchical research approach was structured to identify biomarkers with differential expression in advanced stages of mCPRC compared to initial stages. Lipids identified were then tested in univariate and multivariate models to assess their prognostic effect on OS. This approach is different from other studies that used Cox regression and latent class analysis without initial skimming of potentially interesting lipids. In addition, the 3-lipid signature previously reported requires a formula that is difficult to apply in clinical practice^18^; furthermore, in our cohort, two of three lipids included in this signature, individually analyzed, had no relevant association with patients’ OS. The main limitations of our study are related to the small sample size and the lack of independent validation of C18 Cer, which needs to be assessed in further studies.

## Conclusion

In this study, we explored the prognostic and predictive value of several lipid species in patients with mCRPC by using an untargeted lipidomic approach that combined mass spectrometry techniques with liquid chromatography.

We found that specific CAR and Cer show prognostic significance in patients with mCRPC and could serve as new clinical biomarkers. We uncovered that C18 Cer had independent prognostic capacity in our cohort of patients and was associated with OS after adjustment for relevant confounding factors.

Lipids included in the previously reported 3-lipid signature did not have statistically significant prognostic significance in our cohort, except for ceramide d18:1/24:1, which was only associated with patient OS in univariate analysis.

In light of these results, further studies are needed to validate the prognostic significance of C18 Cer and to explore the predictive value of other ceramides in patients with mCRPC.

Finally, literature data support the notion that targeting sphingolipid metabolism is a feasible approach that could be tested in patients with mCRPC and may lead to the discovery of new active drugs in PCa.

### Supplementary Information

Supplementary Information 1.

Supplementary Tables.

## Data Availability

The datasets generated and analyzed during the current study are not publicly available due to privacy reasons but are available from the corresponding author on reasonable request.
