# Supplementary material for: Untargeted lipidomics reveal association of elevated plasma C18 ceramide levels with reduced survival in metastatic castration-resistant prostate cancer patients
Source: Sci Rep. 2023 Oct 18;13:17791. doi: 10.1038/s41598-023-44157-9 (PMC10585001; doi:10.1038/s41598-023-44157-9)
Supplement: Supplementary file 1 — Supplementary Information 1. [file 41598_2023_44157_MOESM1_ESM.pdf]

## Supplementary File 1

An analysis was conducted on individual lipids exhibiting statistically significant differences in expression between cohorts with 1L and >2L (referring to the discovery lipidomic analysis). These lipids were subject to X-Tile analysis to determine the optimal cutoff values and their correlation with the overall survival of patients. Lipids demonstrating a proportional increase in the relative risk of mortality based on plasma values were subsequently chosen for evaluation in both univariate and multivariate analyses.

| | FC | log2(FC) | raw.pval | Relative Risk Plot | Cut off | RR | RR p-value |
|---|---|---|---|---|---|---|---|
| **CAR 11:1** | 1,3118 | 0,39155 | 0,017884 | 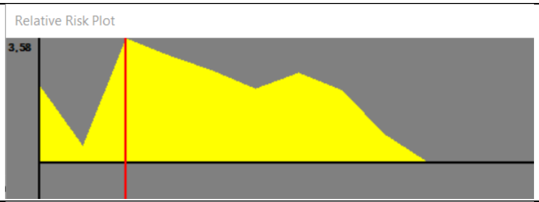 | 4 | 3.58 | 0.00039 |
| **CAR 12:0** | 1,6303 | 0,70517 | 0,02664 | 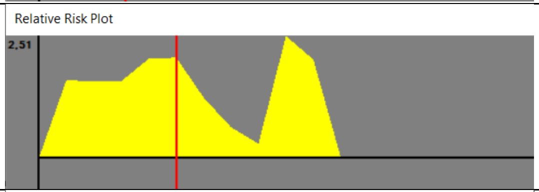 | 6 | 2.51 | 0.021 |
| **CAR 14:0** | 3,7366 | 1,9017 | 0,036801 | 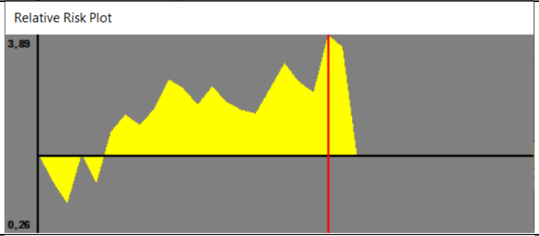 | 5.6 | 3.89 | 0.033 |
| **CAR 18:0** | 2,3402 | 1,2267 | 0,015352 | 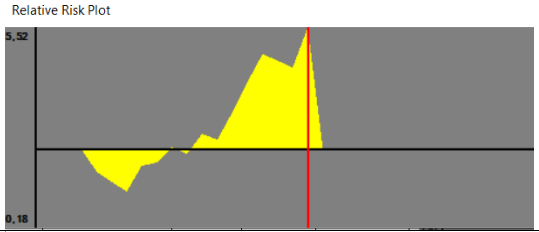 | 5.3 | 5.32 | 0.00078 |
| **CAR 18:1** | 1,8577 | 0,89351 | 0,03911 | 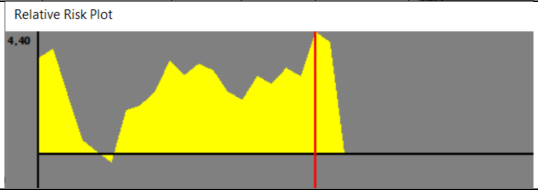 | 5.7 | 4.40 | |

| | | | | | | | |
|---|---|---|---|---|---|---|---|
| **CAR 20:0** | 1,513 | 0,59741 | 0,028126 | 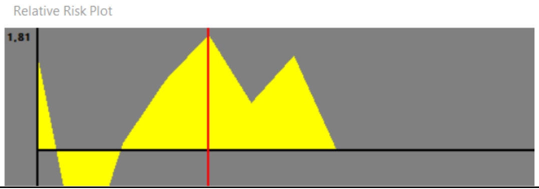 | 6 | 1.81 | 0.19 |
| **CAR 20:1** | 2,6129 | 1,3856 | 0,031974 | 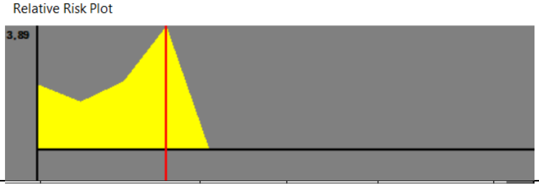 | 5 | 3.89 | 0.028 |
| **CAR 24:1** | 1,6236 | 0,69916 | 0,0011274 | 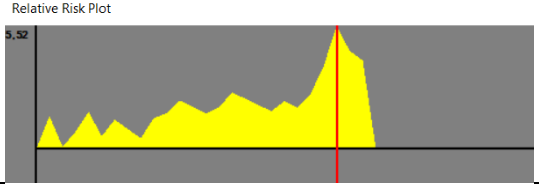 | 6.8 | 3.89 | 0.00964 |
| **CAR 26:1** | 1,327 | 0,40819 | 0,015041 | 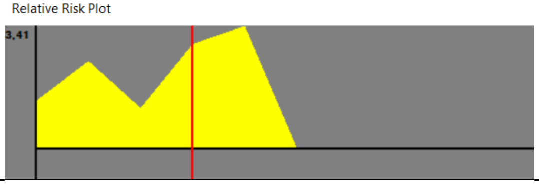 | 6 | 3.41 | 0.0736 |
| **Cer 34:0;2O\|Cer 18:0;2O/16:0** | 1,564 | 0,64525 | 0,012964 | 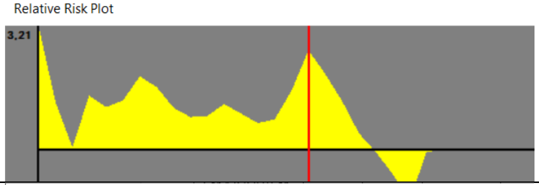 | 34 | 3.21 | 0.138 |
| **Cer 34:1;2O\|Cer 18:1;2O/16:0** | 1,3949 | 0,48011 | 0,00079806 | 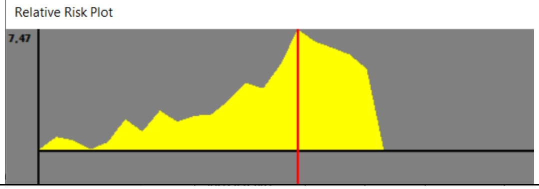 | 34 | 7.47 | 0.00056 |
| **Cer 36:1;2O\|Cer 18:1;2O/18:0** | 1,4895 | 0,57484 | 0,010263 | 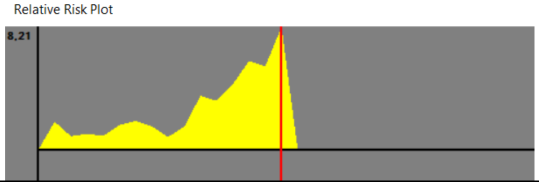 | 30 | 8.21 | 0.00013 |

| | | | | | | | | |
|---|---|---|---|---|---|---|---|---|
| **Cer 36:2;2O\|Cer 18:2;2O/18:0** | 1,6565 | 0,72814 | 0,0041042 | | 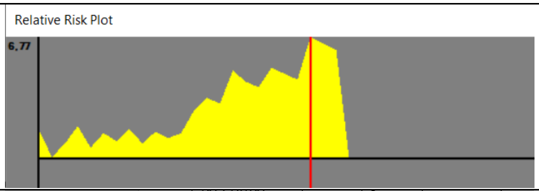 | 30 | 6.77 | 0.0179 |
| **Cer 42:2;2O\|Cer 18:1;2O/24:1** | 1,3712 | 0,45543 | 0,0035211 | | 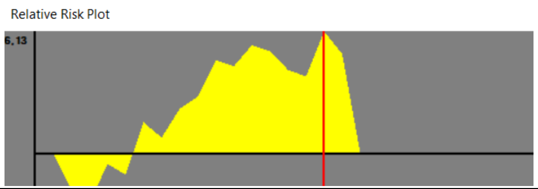 | 31 | 6.13 | 0.0319 |
| **Cer 44:2;2O\|Cer 20:1;2O/24:1** | 1,3794 | 0,46401 | 0,0089739 | | 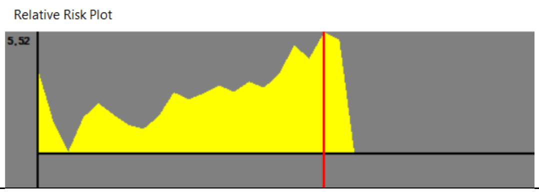 | 33 | 5.52 | 0.00617 |
| **DG 28:2** | 4,1628 | 2,0576 | 0,01439 | | 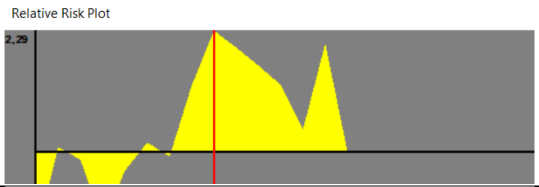 | 14 | 2.29 | 0.37109 |
| **DG 29:4\|DG 11:0_18:4** | 0,63116 | -0,66393 | 0,015955 | | 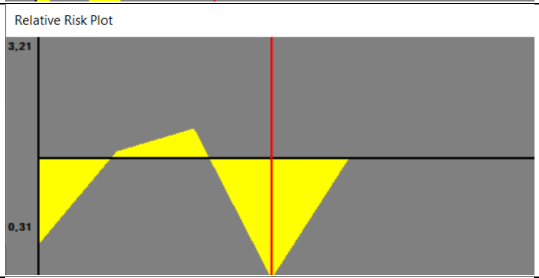 | 3 | 0.31 | 0.317 |
| **DG 34:2** | 1,5074 | 0,59203 | 0,010138 | | 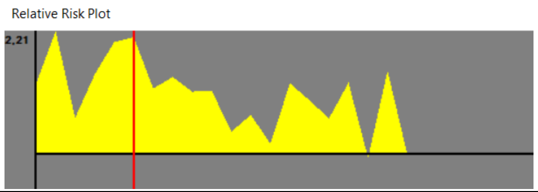 | 1.1 | | |

| | | | | | | | | |
|---|---|---|---|---|---|---|---|---|
| **DG 36:1\|DG 18:0_18:1** | 1,5182 | 0,6024 | 0,02745 | | 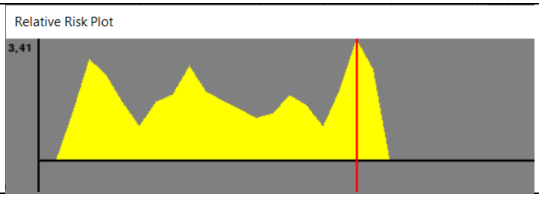 | 2.8 | 0 | 0.75 |
| **DG 37:7** | 0,71118 | -0,49172 | 0,033409 | | 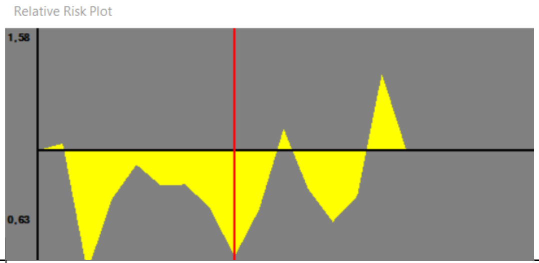 | 1.1 | 0.63 | ? |
| **Hex2Cer 32:1;2O\|Hex2Cer 18:1;2O/14:0** | 0,66182 | -0,59548 | 0,0061158 | | 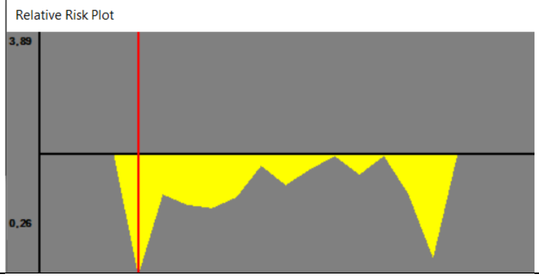 | 0.5 | 0.26 | ? |
| **LPC 18:2/0:0** | 0,70137 | -0,51175 | 0,047975 | | 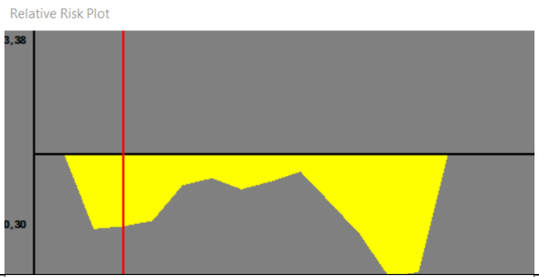 | 3 | 0.30 | 0.0404 |
| **LPE 18:1** | 0,73527 | -0,44365 | 0,048796 | | 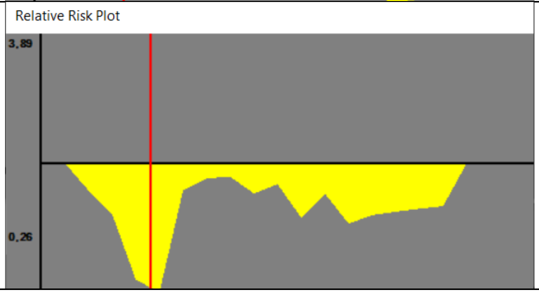 | 0.7 | 0.26 | |

| | | | | | | | | | |
|---|---|---|---|---|---|---|---|---|---|
| **PC 36:4\|PC 18:2_18:2** | 0,60335 | -0,72894 | 0,020285 | | 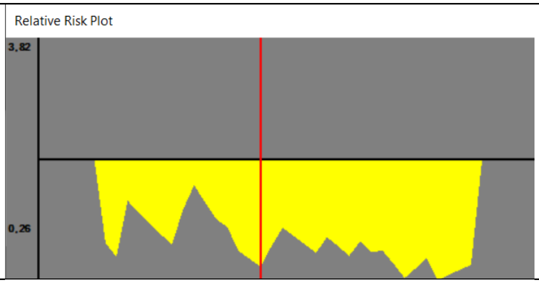 | 43 | 0.26 | 0.179 |
| **PC 36:5\|PC 18:2_18:3** | 0,54818 | -0,86728 | 0,027465 | | 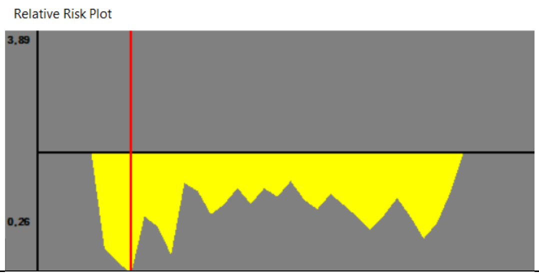 | 10 | 0.26 | 0.00102 |
| **PC 37:2\|PC 19:0_18:2** | 0,75556 | -0,40438 | 0,045237 | | 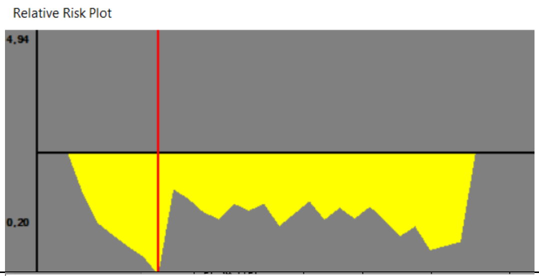 | 20 | 0.20 | 0.00050 |
| **PC O-38:7** | 1,3438 | 0,42627 | 0,016652 | | 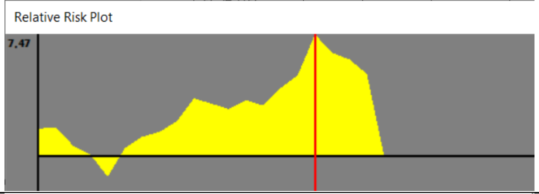 | 31 | 7.47 | 0.10035 |
| **PC O-38:7\|PC O-16:1_22:6** | 1,3547 | 0,43794 | 0,014878 | | 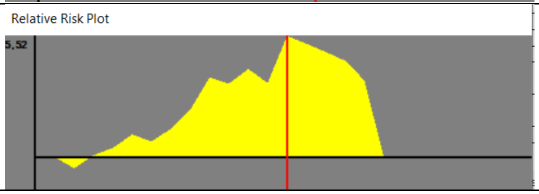 | 29 | 5.52 | 0.3173 |

| | | | | | | | |
|---|---|---|---|---|---|---|---|
| **PC O-39:3** | 0,52201 | -0,93784 | 0,018891 | 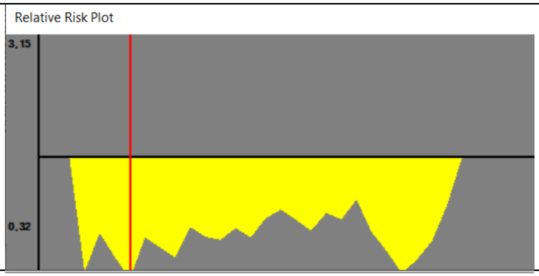 | 13 | 0.32 | 0.00113 |
| **PC O-40:10** | 1,4059 | 0,49152 | 0,04951 | 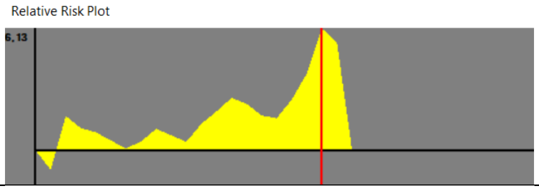 | 38 | 6.13 | 0.138 |
| **PC O-44:8** | 0,60795 | -0,71797 | 0,043982 | 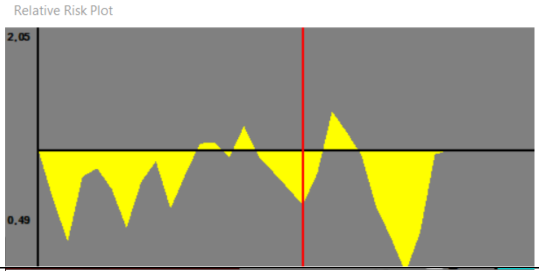 | 37 | 0.49 | 0.179 |
| **PE 34:1\|PE 16:0_18:1** | 1,581 | 0,66088 | 0,0068729 | 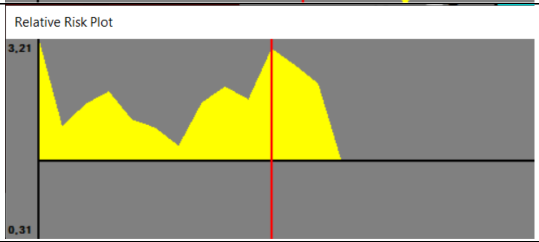 | 1,4 | 3.21 | 0.168 |
| **PE 34:2\|PE 16:0_18:2** | 1,5143 | 0,59866 | 0,016418 | 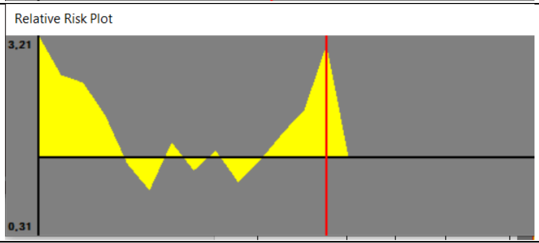 | 1,7 | 3.21 | 0.02259 |

| | | | | | | | | |
|---|---|---|---|---|---|---|---|---|
| **PE 36:2** | 1,4357 | 0,5218 | 0,019561 | | 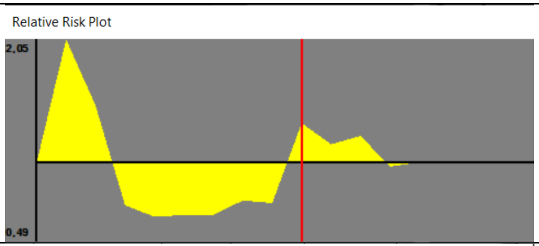 | 1,4 | ? | 0.65 |
| **PE 36:2\|PE 18:0_18:2** | 1,4466 | 0,53269 | 0,038392 | | 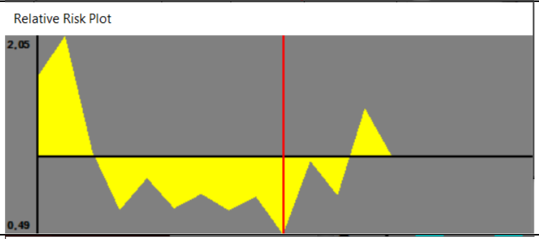 | 1,4 | 0.49 | 0.65 |
| **PE 36:4\|PE 16:0_20:4** | 1,3818 | 0,46655 | 0,039639 | | 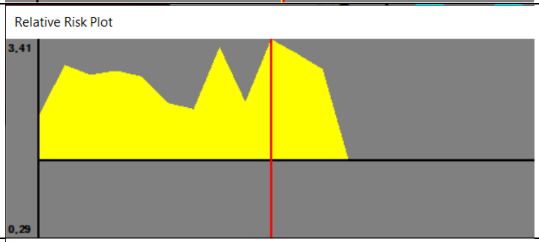 | 1,2 | 3.41 | 0.20 |
| **PE 38:4\|PE 18:0_20:4** | 1,3841 | 0,46895 | 0,0089868 | | 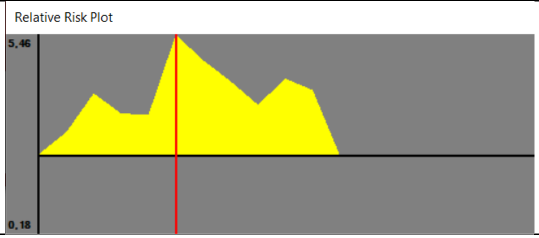 | 0.9 | 5.46 | 0.00964 |
| **PE 38:6\|PE 16:0_22:6** | 1,905 | 0,92982 | 0,00042851 | | 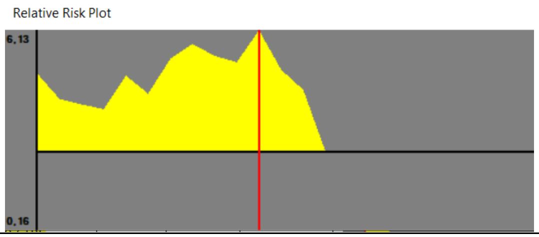 | 1.1 | 6.13 | 0.00617 |

| | | | | | | | | |
|---|---|---|---|---|---|---|---|---|
| **PE 40:6\|PE 18:0_22:6** | 2,2618 | 1,1774 | 9,44E-05 | | 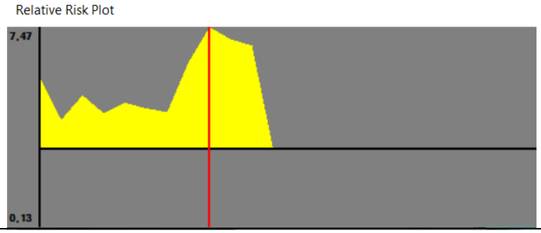 | 0.9 | 7.47 | 0.00205 |
| **PE O-38:7\|PE O-18:2_20:5** | 1,4014 | 0,48684 | 0,0091798 | | 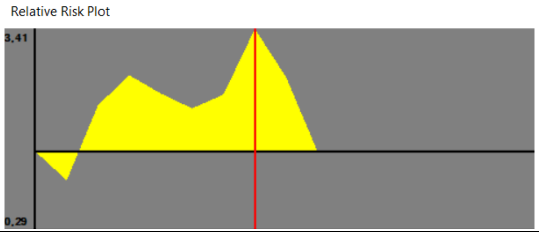 | 1.0 | 3.41 | 0.75 |
| **PE P-38:6\|PE P-16:0_22:6** | 1,3451 | 0,42772 | 0,023263 | | 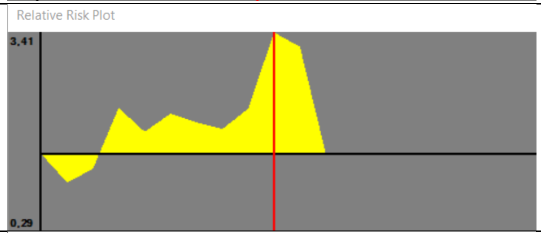 | 1.2 | 3.41 | 0.7518 |
| **SM 30:2;2O** | 0,6865 | -0,54266 | 0,046993 | | 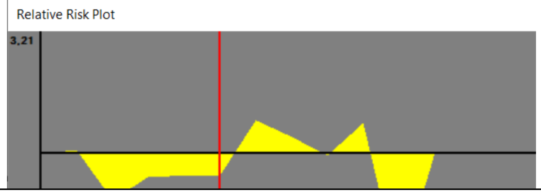 | 5 | 0.1 | 0.06 |
| **SM 34:0;2O\|SM 10:0;2O/24:0** | 1,3156 | 0,39569 | 0,042839 | | 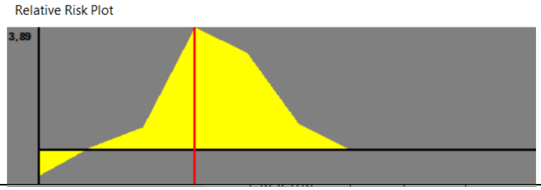 | 5 | 3.89 | 0.2367 |
| **SM 36:0;2O\|SM 26:0;2O/10:0_SM 36:0;2O** | 1,6239 | 0,6995 | 0,010437 | | 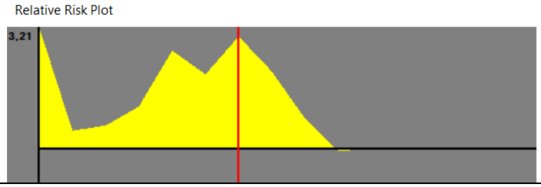 | 7 | 3.21 | 0.05125 |

| | | | | | | | | |
|---|---|---|---|---|---|---|---|---|
| **SM 36:0;2O\|SM 9:0;2O/27:0** | 1,6179 | 0,69413 | 0,010008 | 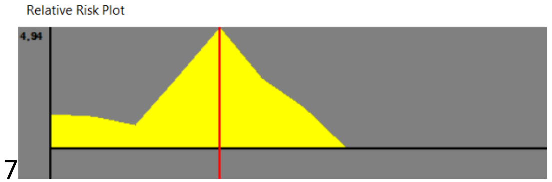 | 5 | 4.94 | 0.1135 |
| **SM 37:1;2O\|SM 27:1;2O/10:0** | 1,355 | 0,43828 | 0,020679 | 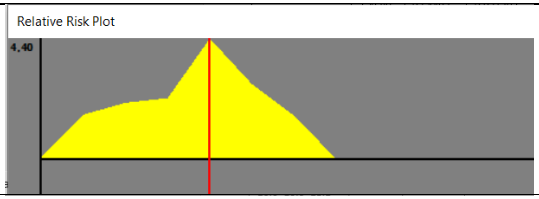 | 5 | 4.40 | 0.00020 |
| **SM 42:2;2O** | 1,4538 | 0,5398 | 0,038143 | 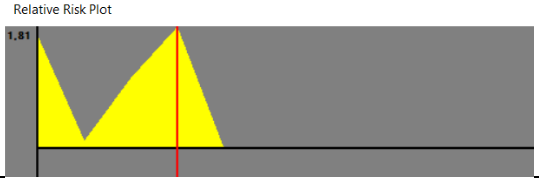 | 5 | 1.81 | 0.25421 |
| **ST 29:1;O;S** | 0,54664 | -0,87134 | 0,013015 | 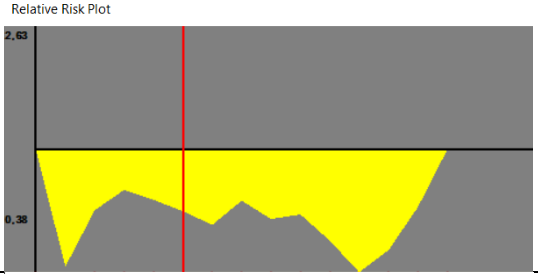 | 5 | 0.38 | 0.04550 |
| **TG 49:0\|TG 15:0_16:0_18:0** | 1,3836 | 0,46839 | 0,039193 | 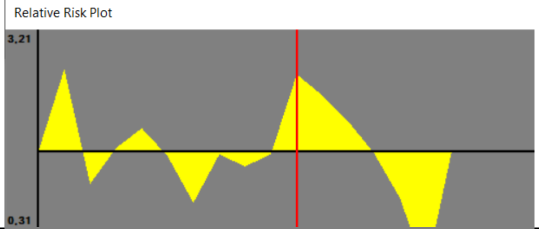 | 13 | 3.21 | 0.27332 |
| **TG 50:0\|TG 14:0_16:0_20:0** | 1,4589 | 0,54492 | 0,021797 | 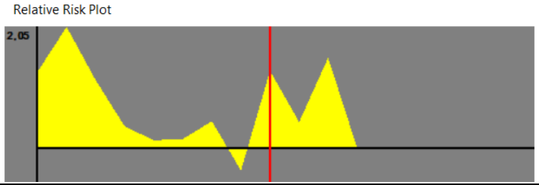 | 10 | 2.05 | 0.58388 |

| | | | | | | | |
|---|---|---|---|---|---|---|---|
| **TG 50:0\|TG 16:0_16:0_18:0** | 1,54 | 0,62298 | 0,021297 | 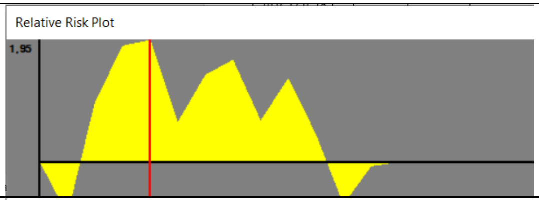 | 5 | 1.95 | 0.27332 |
| **TG 50:1\|TG 16:0_16:0_18:1** | 1,544 | 0,62666 | 0,015135 | 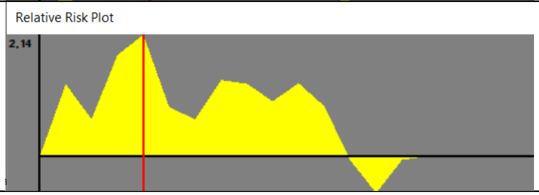 | 4 | 2.14 | 0.19229 |
| **TG 50:2\|TG 16:0_16:1_18:1** | 1,5069 | 0,59156 | 0,030635 | 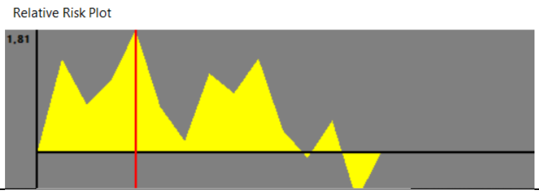 | 5 | 1.81 | 0.37109 |
| **TG 51:0\|TG 16:0_17:0_18:0** | 1,3583 | 0,44175 | 0,019295 | 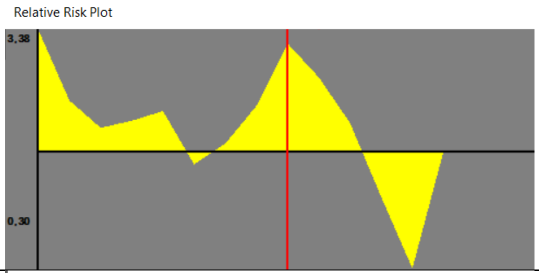 | 13 | 3.38 | 0.19229 |
| **TG 51:1\|TG 16:0_17:0_18:1** | 1,5381 | 0,62118 | 0,033325 | 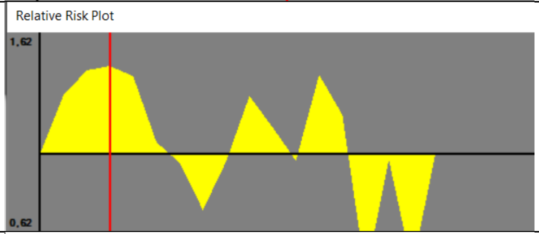 | 4 | 1.62 | 0.37109 |
| **TG 51:2\|TG 16:0_17:1_18:1** | 1,4691 | 0,55493 | 0,04823 | 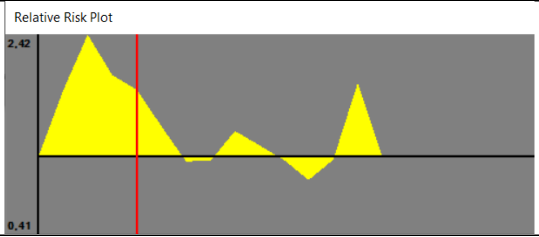 | 4 | 2.42 | 0.276332 |

| | | | | | | | |
|---|---|---|---|---|---|---|---|
| **TG 52:0\|TG 16:0_18:0_18:0** | 1,5814 | 0,66117 | 0,043222 | 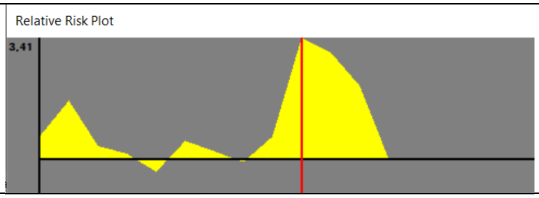  | 11 | 3.41 | 0.52709 |
| **TG 52:1\|TG 16:0_18:0_18:1** | 1,5492 | 0,63157 | 0,014998 | 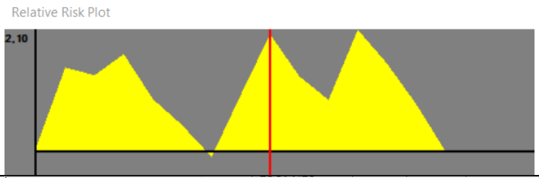  | 10 | 2.10 | 0.65472 |
| **TG 52:2\|TG 16:0_18:1_18:1** | 1,4288 | 0,5148 | 0,019798 | 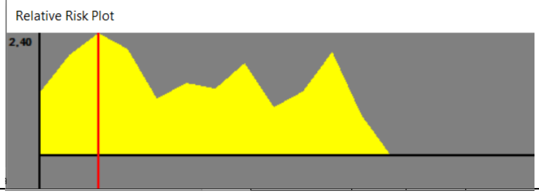  | 4 | 2.40 | 0.106 |
| **TG 52:3\|TG 16:0_18:1_18:2** | 1,4105 | 0,49625 | 0,027501 | 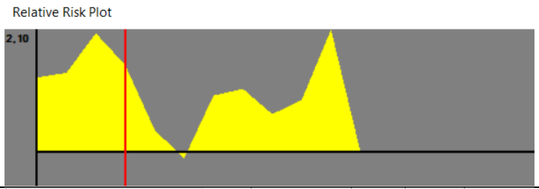  | 5 | 2.10 | 0.34270 |
| **TG 53:0\|TG 14:0_15:0_24:0** | 1,3014 | 0,38007 | 0,0040493 | 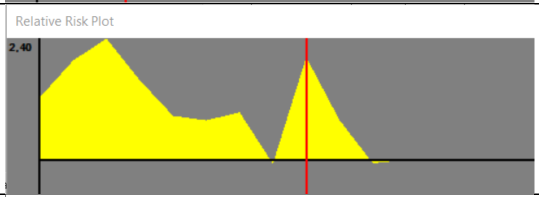  | 13 | 2.40 | 0.254 |
| **TG 53:1\|TG 17:0_18:0_18:1** | 1,4933 | 0,57847 | 0,033677 | 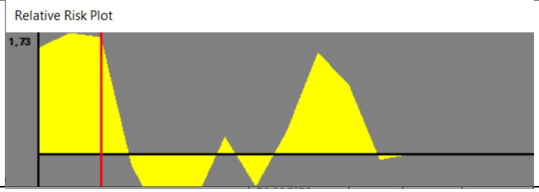  | 4 | 1.73 | 0.2367 |
| **TG 54:1\|TG 18:0_18:0_18:1** | 1,4149 | 0,50066 | 0,026565 | 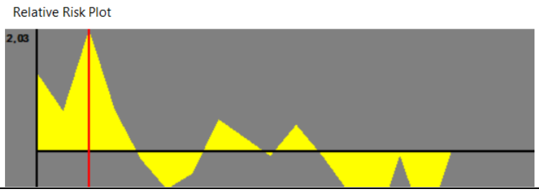  | 4 | 2.03 | 0.11 |

| | | | | | | | |
|---|---|---|---|---|---|---|---|
| **TG 55:1\|TG 18:0_19:0_18:1** | 1,3967 | 0,48198 | 0,043467 | 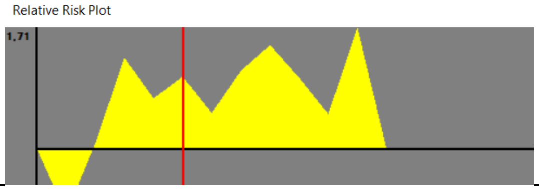  | 6 | 1.71 | 0.751 |
| **TG 56:5\|TG 18:0_18:1_20:4** | 1,3321 | 0,41369 | 0,016882 | 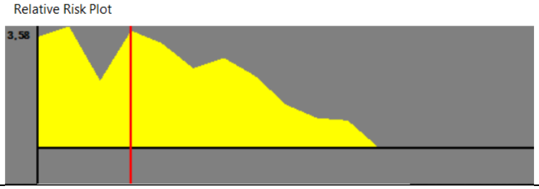  | 5 | 3.58 | 0.03811 |
| **TG 56:6\|TG 16:0_18:1_22:5** | 1,3546 | 0,43784 | 0,027813 | 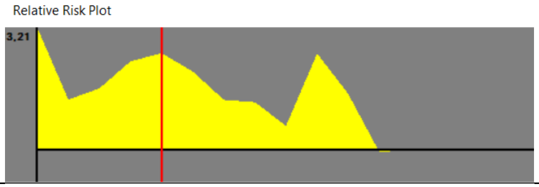  | 6 | 3.21 | 0.13801 |
| **TG 56:7\|TG 16:0_18:1_22:6** | 1,7521 | 0,80912 | 0,030021 | 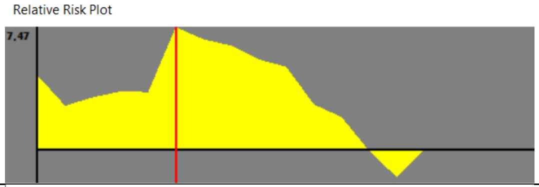  | 6 | 7.47 | 0.05125 |
| **TG 56:7\|TG 18:1_18:2_20:4** | 1,7779 | 0,83018 | 0,025153 | 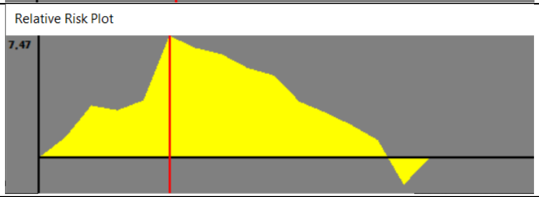  | 6 | 7.47 | 0.05125 |
