# Supplementary material for: Untargeted lipidomics reveal association of elevated plasma C18 ceramide levels with reduced survival in metastatic castration-resistant prostate cancer patients
Source: Sci Rep. 2023 Oct 18;13:17791. doi: 10.1038/s41598-023-44157-9 (PMC10585001; doi:10.1038/s41598-023-44157-9)
Supplement: Supplementary file 2 — Supplementary Tables. [file 41598_2023_44157_MOESM2_ESM.docx]

Table 1 SUPP list of 63 overexpressed lipids in >2 l compared to 1 l patients, based on fold change (FC).

Table shows FC, log2(FC) and p value. DG = diacylglycerol; CAR = carnitine; PC = phosphatidylcholine; PE = phosphatidylethanolamine; TG = triacylglycerol; Cer = ceramide; SM = sphingomyelin.

| **LIPID SPECIES** | **FC** | **log2(FC)** | **p value** |
| --- | --- | --- | --- |
| DG 28:2 | 4,1628 | 2,0576 | 0,01439 |
| CAR 14:0 | 3,7366 | 1,9017 | 0,036801 |
| CAR 20:1 | 2,6129 | 1,3856 | 0,031974 |
| CAR 18:0 | 2,3402 | 1,2267 | 0,015352 |
| PE 40:6\|PE 18:0_22:6 | 2,2618 | 1,1774 | 9,44E-05 |
| PE 38:6\|PE 16:0_22:6 | 1,905 | 0,92982 | 0,000429 |
| CAR 18:1 | 1,8577 | 0,89351 | 0,03911 |
| TG 56:7\|TG 18:1_18:2_20:4 | 1,7779 | 0,83018 | 0,025153 |
| TG 56:7\|TG 16:0_18:1_22:6 | 1,7521 | 0,80912 | 0,030021 |
| Cer 36:2;2O\|Cer 18:2;2O/18:0 | 1,6565 | 0,72814 | 0,004104 |
| CAR 12:0 | 1,6303 | 0,70517 | 0,02664 |
| SM 36:0;2O\|SM 26:0;2O/10:0_SM 36:0;2O | 1,6239 | 0,6995 | 0,010437 |
| CAR 24:1 | 1,6236 | 0,69916 | 0,001127 |
| SM 36:0;2O\|SM 9:0;2O/27:0 | 1,6179 | 0,69413 | 0,010008 |
| TG 52:0\|TG 16:0_18:0_18:0 | 1,5814 | 0,66117 | 0,043222 |
| PE 34:1\|PE 16:0_18:1 | 1,581 | 0,66088 | 0,006873 |
| Cer 34:0;2O\|Cer 18:0;2O/16:0 | 1,564 | 0,64525 | 0,012964 |
| TG 52:1\|TG 16:0_18:0_18:1 | 1,5492 | 0,63157 | 0,014998 |
| TG 50:1\|TG 16:0_16:0_18:1 | 1,544 | 0,62666 | 0,015135 |
| TG 50:0\|TG 16:0_16:0_18:0 | 1,54 | 0,62298 | 0,021297 |
| TG 51:1\|TG 16:0_17:0_18:1 | 1,5381 | 0,62118 | 0,033325 |
| DG 36:1\|DG 18:0_18:1 | 1,5182 | 0,6024 | 0,02745 |
| PE 34:2\|PE 16:0_18:2 | 1,5143 | 0,59866 | 0,016418 |
| CAR 20:0 | 1,513 | 0,59741 | 0,028126 |
| DG 34:2 | 1,5074 | 0,59203 | 0,010138 |
| TG 50:2\|TG 16:0_16:1_18:1 | 1,5069 | 0,59156 | 0,030635 |
| TG 53:1\|TG 17:0_18:0_18:1 | 1,4933 | 0,57847 | 0,033677 |
| **Cer 36:1;2O\|Cer 18:1;2O/18:0** | 1,4895 | 0,57484 | 0,010263 |
| TG 51:2\|TG 16:0_17:1_18:1 | 1,4691 | 0,55493 | 0,04823 |
| TG 50:0\|TG 14:0_16:0_20:0 | 1,4589 | 0,54492 | 0,021797 |
| SM 42:2;2O | 1,4538 | 0,5398 | 0,038143 |
| PE 36:2\|PE 18:0_18:2 | 1,4466 | 0,53269 | 0,038392 |
| PE 36:2 | 1,4357 | 0,5218 | 0,019561 |
| TG 52:2\|TG 16:0_18:1_18:1 | 1,4288 | 0,5148 | 0,019798 |
| TG 54:1\|TG 18:0_18:0_18:1 | 1,4149 | 0,50066 | 0,026565 |
| TG 52:3\|TG 16:0_18:1_18:2 | 1,4105 | 0,49625 | 0,027501 |
| PC O-40:10 | 1,4059 | 0,49152 | 0,04951 |
| PE O-38:7\|PE O-18:2_20:5 | 1,4014 | 0,48684 | 0,00918 |
| TG 55:1\|TG 18:0_19:0_18:1 | 1,3967 | 0,48198 | 0,043467 |
| Cer 34:1;2O\|Cer 18:1;2O/16:0 | 1,3949 | 0,48011 | 0,000798 |
| PE 38:4\|PE 18:0_20:4 | 1,3841 | 0,46895 | 0,008987 |
| TG 49:0\|TG 15:0_16:0_18:0 | 1,3836 | 0,46839 | 0,039193 |
| PE 36:4\|PE 16:0_20:4 | 1,3818 | 0,46655 | 0,039639 |
| Cer 44:2;2O\|Cer 20:1;2O/24:1 | 1,3794 | 0,46401 | 0,008974 |
| **Cer 42:2;2O\|Cer 18:1;2O/24:1** | 1,3712 | 0,45543 | 0,003521 |
| TG 51:0\|TG 16:0_17:0_18:0 | 1,3583 | 0,44175 | 0,019295 |
| SM 37:1;2O\|SM 27:1;2O/10:0 | 1,355 | 0,43828 | 0,020679 |
| PC O-38:7\|PC O-16:1_22:6 | 1,3547 | 0,43794 | 0,014878 |
| TG 56:6\|TG 16:0_18:1_22:5 | 1,3546 | 0,43784 | 0,027813 |
| PE P-38:6\|PE P-16:0_22:6 | 1,3451 | 0,42772 | 0,023263 |
| PC O-38:7 | 1,3438 | 0,42627 | 0,016652 |
| TG 56:5\|TG 18:0_18:1_20:4 | 1,3321 | 0,41369 | 0,016882 |
| CAR 26:1 | 1,327 | 0,40819 | 0,015041 |
| SM 34:0;2O\|SM 10:0;2O/24:0 | 1,3156 | 0,39569 | 0,042839 |
| CAR 11:1 | 1,3118 | 0,39155 | 0,017884 |
| TG 53:0\|TG 14:0_15:0_24:0 | 1,3014 | 0,38007 | 0,004049 |
| TG 58:0\|TG 16:0_17:0_25:0 | 1,282 | 0,35834 | 0,030393 |
| TG 56:0\|TG 15:0_16:0_25:0 | 1,2726 | 0,34775 | 0,010269 |
| PC 33:0\|PC 16:0_17:0 | 1,2607 | 0,33421 | 0,019922 |
| TG 54:0\|TG 16:0_18:0_20:0 | 1,2606 | 0,33407 | 0,019337 |
| TG 57:0\|TG 16:0_17:0_24:0 | 1,2369 | 0,30674 | 0,024409 |
| TG 56:4\|TG 18:0_18:1_20:3 | 1,228 | 0,29626 | 0,041971 |
| SM 36:2;2O\|SM 16:1;2O/20:1 | 1,2073 | 0,2718 | 0,03924 |

Table 2 SUPP list of 12 underexpressed lipids in >2 l compared to 1 l patients, based on fold change (FC).

Table shows FC, log2(FC) and p value. DG= diacylglycerol; Hex2Cer= dihexosylceramide; LPC=lysophosphatidylcholine; LPE=lysophosphatidylethanolamine; PC: phosphatidylcholine; PE = phosphatidylethanolamine; SM = sphingomyelin; ST= sterols.

| **LIPID SPECIES** | **FC** | **log2(FC)** | **p value** |
| --- | --- | --- | --- |
| PC O-39:3 | 0,52201 | -0,93784 | 0,018891 |
| ST 29:1;O;S | 0,54664 | -0,87134 | 0,013015 |
| PC 36:5\|PC 18:2_18:3 | 0,54818 | -0,86728 | 0,027465 |
| PC 36:4\|PC 18:2_18:2 | 0,60335 | -0,72894 | 0,020285 |
| PC O-44:8 | 0,60795 | -0,71797 | 0,043982 |
| DG 29:4\|DG 11:0_18:4 | 0,63116 | -0,66393 | 0,015955 |
| Hex2Cer 32:1;2O\|Hex2Cer 18:1;2O/14:0 | 0,66182 | -0,59548 | 0,006116 |
| SM 30:2;2O | 0,6865 | -0,54266 | 0,046993 |
| LPC 18:2/0:0 | 0,70137 | -0,51175 | 0,047975 |
| DG 37:7 | 0,71118 | -0,49172 | 0,033409 |
| LPE 18:1 | 0,73527 | -0,44365 | 0,048796 |
| PC 37:2\|PC 19:0_18:2 | 0,75556 | -0,40438 | 0,045237 |
| DG 30:6 | 0,79132 | -0,33766 | 0,027953 |
